# Association rule analysis of food-specific IgG and clinical symptoms in pediatric patients

**DOI:** 10.3389/fimmu.2026.1940230

**Published:** 2026-09-16

**Authors:** Zidong Zhou, Hongqiu Wu, Xifeng Qiao

**Affiliations:** 1 YunKang School of Medicine and Health, Nanfang College Guangzhou, Guangzhou, Guangdong, China; 2 Department of Clinical Laboratory, Zhuhai Hospital of Traditional Chinese Medicine, Zhuhai, Guangdong, China

**Keywords:** association rule mining, children, clinical symptoms, food intolerance, food-specific IgG

## Abstract

Food-specific immunoglobulin G (IgG) have drawn increasing attention in pediatric food intolerance study, yet population-based data and their associations with clinical symptoms remain limited. This retrospective study included 100 children (56 males, 44 females; age groups: <5 years, n=20; 5-9 years, n=50; ≥10 years, n=30) from Zhuhai, China. Serum specific IgG against 14 common foods were measured by enzyme-linked immunosorbent assay (ELISA) and classified as negative (<50 U/mL), mild (50-100 U/mL), moderate (100-200 U/mL), or severe (≥200 U/mL). Spearman correlation and Apriori association rule analysis (minimum support 6%, minimum confidence 50%, lift >1.5) were applied. The three most prevalent food-specific IgG positivities were egg (89.0%), milk (73.0%), and wheat (48.0%). Total positivity rate of each food correlated positively with its severe proportion (r=0.8733, P<0.001). No significant differences between sex were found in positivity rates, but the correlation patterns differed. In boys, both moderate and severe proportions correlated with positivity rates, while in girls, only the severe proportion did. Age showed no significant effect on positivity rates, though food-specific IgG response intensity trend to decrease with age. The four most frequent clinical symptoms were cough-related (25 cases), nasal diseases (20 cases), tic disorder/chronic tic disorders (19 cases), and deficiency-excess complex pattern (9 cases). Association rule mining identified four strong rules; the strongest was deficiency-excess complex pattern with egg and milk co-positivity (support 7.0%, confidence 77.8%, lift 6.09). These findings suggest that egg, milk, and wheat are the predominant IgG‑positive food items in this cohort. The positive correlation between positivity rate and severe proportion, along with the strong association between deficiency-excess complex pattern and egg-milk co-positivity, may inform dietary interventions in children with food intolerance, though prospective validation is warranted.

## Introduction

Food intolerance (FI) is a delayed immune response mediated by food-specific immunoglobulin G (IgG), classified as a type III hypersensitivity reaction. Its pathogenesis primarily involves impaired intestinal barrier function, translocation of incompletely digested food macromolecular antigens into the circulation, and loss of immune tolerance to food antigens (1). Unlike IgE-mediated immediate-type allergic reactions, IgG-mediated FI has an insidious onset with a latency of several hours to days, and the inflammatory response is non-specific and diffusely distributed (2). Children are particularly susceptible to FI due to their relatively high intestinal mucosal permeability, insufficient digestive enzyme secretion, and immature intestinal immune system. Following exposure to intolerant food antigens, this population may present with a wide spectrum of clinical manifestations, including gastrointestinal symptoms such as diarrhea, abdominal pain, and dyspepsia, as well as involvement of the skin, respiratory, and nervous systems, manifesting as eczema, chronic cough, attention deficits, and migraines, which are often overlooked or misdiagnosed.

In recent years, the clinical significance of food-specific IgG4 (FS-IgG4), a major IgG subclass produced in response to food antigen exposure, has drawn increasing attention. A multicenter study involving 407 children with allergic diseases demonstrated that dietary avoidance guided by FS-IgG4 testing significantly improved clinical symptoms, and the decrease in FS-IgG4 levels after treatment independently predicted symptom remission (3). Among FS-IgG4-positive foods, egg and cow’s milk showed the highest detection rates, which may be attributed to early and sustained exposure to these two foods during childhood (3). However, food-specific IgG4 should be regarded as a normal physiological response to food exposure rather than a pathogenic factor for hypersensitivity.

Among the diverse clinical symptoms associated with FI, allergic rhinitis, chronic cough, and tic disorders are characterized by prolonged disease course, poor response to conventional treatment, and strong links to FI-related chronic inflammation. The incidence of these three chronic conditions in children has been rising in recent years, adversely affecting patients’ quality of life and mental health. Circulating immune complexes formed by food-specific IgG antibodies can deposit in multiple organ systems, triggering persistent low-grade systemic inflammation (4), which may partially explain the limited efficacy of conventional therapies for these conditions. The elucidation of the gut-lung axis and gut-brain axis mechanisms has provided a theoretical framework for understanding how food antigens derived from the gut affect distal organs (5). Therefore, identifying and eliminating potential dietary triggers is of great significance for the comprehensive prevention and treatment of chronic diseases in children (6).

Despite the growing recognition of FI’s clinical value, the geographical coverage of relevant studies remains insufficient, with a notable scarcity of regional data. Most epidemiological surveys in China have focused on northern regions such as Hebei and Beijing (7), whereas the food intolerance profile of children in the coastal city-characterized by its unique humid subtropical climate, dietary habits, and genetic background-remains largely unknown. Furthermore, previous studies have predominantly employed univariate or regression analyses, which only allow examination of linear associations between single foods and single diseases, and are inadequate for capturing the complex relationships between multiple food intolerance combinations and diverse clinical symptoms. Indeed, the clustering and co-occurrence of food antigens have been documented (8), highlighting the need for novel analytical approaches to explore multi-food sensitization patterns. In addition, the influence of demographic factors such as sex and age on FI positivity rates and food-specific IgG response intensity requires further elucidation.

To address these gaps, the present study enrolled 100 children under 14 years of age from the coastal city of Zhuhai, measured serum-specific IgG antibodies against 14 common foods, described the positivity rate spectrum and food-specific IgG response intensity distribution of childhood in this region, analyzed their associations with sex and age, and introduced association rule mining to explore the relationship between major food-specific IgG combinations and core clinical symptoms. Our findings may provide a reference for etiological differentiation and individualized dietary intervention in chronic childhood diseases in this region.

## Results

### Demographic and clinical characteristics of the study population

A total of 100 children were enrolled in this study, including 56 males (56.0%) and 44 females (44.0%). The age ranged from 2.0 to 12.8 years, with a mean age of 7.2 ± 2.8 years. The largest age group was 5-9 years, comprising 50 cases (50.0%), followed by the ≥10 years group with 30 cases (30.0%), and the <5 years group with 20 cases (20.0%) (**Table 1**).

**Table 1:** Demographic and clinical characteristics of the 100 study children.

Characteristic	Category	No. of cases (n)	Percentage (%)
Sex	Male	56	56.0
	Female	44	44.0
Age group	<5 years	20	20.0
	5-9 years	50	50.0
	≥10 years	30	30.0
Age (mean ± SD)	-	7.2 ± 2.8	-
Major clinical symptoms	Cough-related	25	25.0
	Nasal diseases	20	20.0
	Tic disorder	19	19.0
	Deficiency-excess complex pattern	9	9.0
	Asthma	4	4.0
	Others (digestive symptoms, fever, etc.)	23	23.0

Clinical symptoms involved 44 different diagnostic labels. After consolidation, the four most frequent symptom categories were: cough-related symptoms in 25 cases (25.0%), including cough, bronchitis, acute bronchitis, cough variant asthma, and *Mycoplasma pneumoniae* acute bronchitis; nasal diseases in 20 cases (20.0%), including allergic rhinitis, sinusitis, nasal obstruction (Bi Qiu), chronic sinusitis, nasal septum deviation, and acute rhinitis with nasal congestion; tic disorder/chronic tic disorders in 19 cases (19.0%), including tic disorder and chronic tic disorders; and deficiency-excess complex pattern in 9 cases (9.0%), including, as well as asthma in 4 cases. The remaining symptoms, including 4 presented with lung-spleen qi deficiency (4 cases), spleen deficiency and dampness retention complicated by phlegm-dampness obstructing the lung (3 cases), and classical mixed deficiency-excess syndrome (2 cases), dyspepsia (4 cases), fever (4 cases), and adenoid hypertrophy, eczema, urticaria, short stature, abdominal pain, constipation, refractive error, nightmare, favism, and angioneurotic edema (1-2 cases each), accounted for 23 cases (23.0%) in total (**Table 1**).

### Higher total positivity rate associated with higher severe proportion

Among the 100 children, the distribution of food-specific IgG positivity rates showed that egg (89.0%), milk (73.0%), and wheat (48.0%) ranked the top three, constituting the primary sources of food-specific IgG in this cohort. Soybean (23.0%) and codfish (10.0%) were in the middle range, while the remaining nine foods were ≤5.0%. The classification was based on the natural distribution of total positivity rates across the 14 foods. Clear numerical gaps were observed: egg (89%), milk (73%), and wheat (48%) were substantially higher than soybean (23%) and codfish (10%), while the remaining nine foods all showed rates ≤5%. Using these natural breakpoints as thresholds, we defined the high-positivity tier as foods with rates ≥40% (egg, milk, wheat), the moderate-positivity tier as foods with rates between 10% and 25% (soybean and codfish), and the low-positivity tier as foods with rates ≤5% (the other nine foods) (**Supplementary Table S1**; **Figure 1A**).

**Figure 1:**
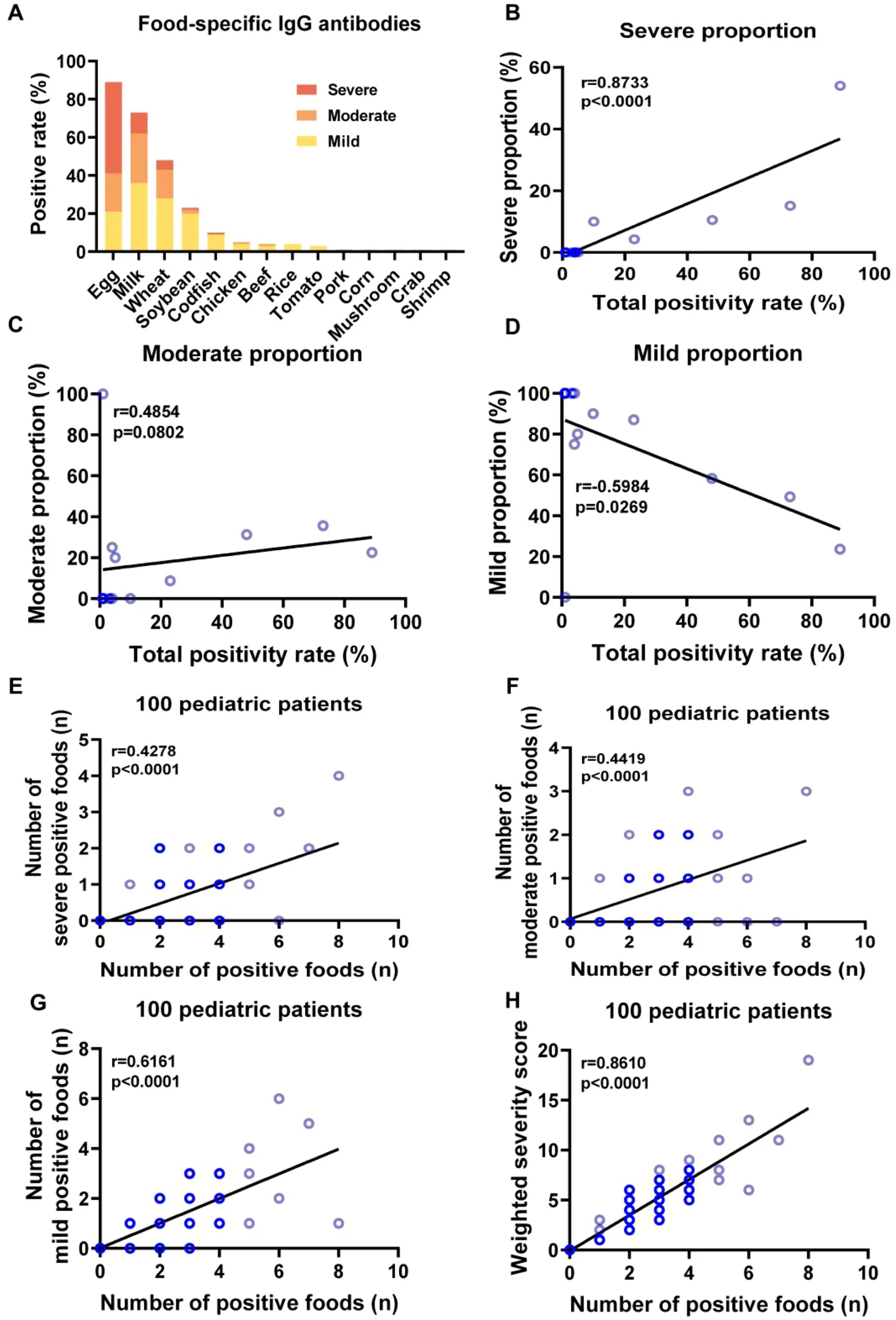
Food-specific IgG antibody profiles and correlation analysis. **(A)** Horizontal stacked bar chart showing total positivity rate and the proportions of mild, moderate, and severe positive cases for each food. Foods are arranged in descending order of total positivity rate. Color segments: mild (+1, 50-100 U/mL), moderate (+2, 100-200 U/mL), and severe (+3, ≥200 U/mL). **(B-D)** Scatter plots showing the correlation between total positivity rate and the proportions of severe **(B)**, moderate **(C)**, and mild **(D)** positive cases among all positive cases for each food. Each dot represents one food item. Spearman correlation coefficients (r) and P values are shown in each panel. The X-axis ranges from 0% to 100%, with the actual positivity rates in this study ranging from 1% (shrimp and crab) to 89% (egg). The Y-axis also ranges from 0% to 100%. **(E-H)** Scatter plots showing the correlations between the number of positive foods per child (x-axis, range 0-14) and four severity indicators (y-axis): number of severe positive foods **(E)**, number of moderate positive foods **(F)**, number of mild positive foods **(G)**, and weighted severity score (mild = +1, moderate = +2, severe = +3; range 0-42) **(H)**. Each dot represents one child (n = 100). Spearman correlation coefficients (r) and P values are shown in each panel.

Regarding food-specific IgG response intensity distribution, foods with higher positivity rates (egg, milk, and wheat) showed considerably higher proportions of moderate and severe immune responses among their positive cases than foods with lower positivity rates. The proportion of moderate and severe immune responses was 76.4% (68/89) for egg, among which severe immune response cases accounted for 53.9% (48/89); 50.7% (37/73) for milk; and 41.7% (20/48) for wheat. In contrast, foods in the moderate- and low-positivity rates predominantly associated with mild immune response. Apart from soybean (2 moderate and 1 severe cases), the remaining low-positivity rate foods rarely induced moderate-to-severe immune responses (**Supplementary Table S1**; **Figure 1A**). Among the 65 children with two or more positive foods, only 12 (18.5%) showed all positive foods clustered within a single severity grade, while the remaining 53 (81.5%) exhibited varying severity levels across different positive foods-for example, severe reactivity to egg, moderate to milk, and mild to wheat. This suggests that, within the same child, the intensity of IgG responses varies across foods, indicating a food-specific immune response pattern rather than a uniform high- or low-responder phenotype. This finding suggests that dietary interventions should assess the response intensity to each positive food individually, rather than applying a uniform strategy to all positive foods in the same child.

At the food level, to further explore the association between positivity rate and food-specific IgG response intensity, we performed Spearman correlation analyses using the total positivity rate of each of the 14 foods as the x-axis and the proportions of mild, moderate, and severe positive cases as the y-axes (**Figures 1B–D**). The results showed that total positivity rate was significantly positively correlated with severe proportion (r = 0.8733, P < 0.001), not correlated with moderate proportion (r = 0.4854, P = 0.0820), and significantly negatively correlated with mild proportion (r = -0.5984, P = 0.0269) (**Figures 1B–D**). These findings suggest that foods with higher total positivity rates tend to have higher proportions of severe reactions and lower proportions of mild reactions among their positive cases, indicating a positive correlation between the breadth (total positivity rate) and depth (severe proportion) of food-specific IgG.

At the individual level, the number of positive foods per child (range 0-14) was positively correlated with the number of severe positive foods (r = 0.4278, P < 0.0001), as well as with the numbers of mild and moderate positive foods (r = 0.6161 and 0.4419, respectively; both P < 0.0001). The strongest correlation was observed between the number of positive foods and the weighted positivity score (mild = +1, moderate = +2, severe = +3; range 0-42) (r = 0.8610, P < 0.0001) (**Figures 1E–H**). This indicates that children with more positive foods tend to have more positive foods across all severity grades, reflecting a heavier overall intolerance burden.

In summary, a positive correlation between the breadth and depth of food-specific IgG was observed at both the food level and the individual level, providing evidence from different dimensions that higher total positivity rates are associated with a higher proportion of severe immune responses.

### Sex does not affect food positivity rates, but the correlation between positivity rate and severe proportion differs by sex

Among the 100 children, 56 were male and 44 were female. No significant differences were observed between sexes in the positivity rates of the 14 food-specific IgG antibodies (all P > 0.05) (**Supplementary Table S1**; **Figure 2A**), suggesting a balanced sex distribution of food-specific IgG in this population. Egg positivity ranked first in both sexes (91.1% in males vs. 86.4% in females), with no statistically significant difference (χ^2^ = 0.558, P = 0.455). Milk ranked second (78.6% in males vs. 65.9% in females), also without significant difference (χ^2^ = 2.005, P = 0.157). Wheat positivity was similar between sexes (46.4% in males vs. 50.0% in females; χ^2^ = 0.126, P = 0.723). No significant sex differences were found for soybean, codfish, or meat products (P > 0.05) (**Supplementary Table S2**; **Figure 2A**).

**Figure 2:**
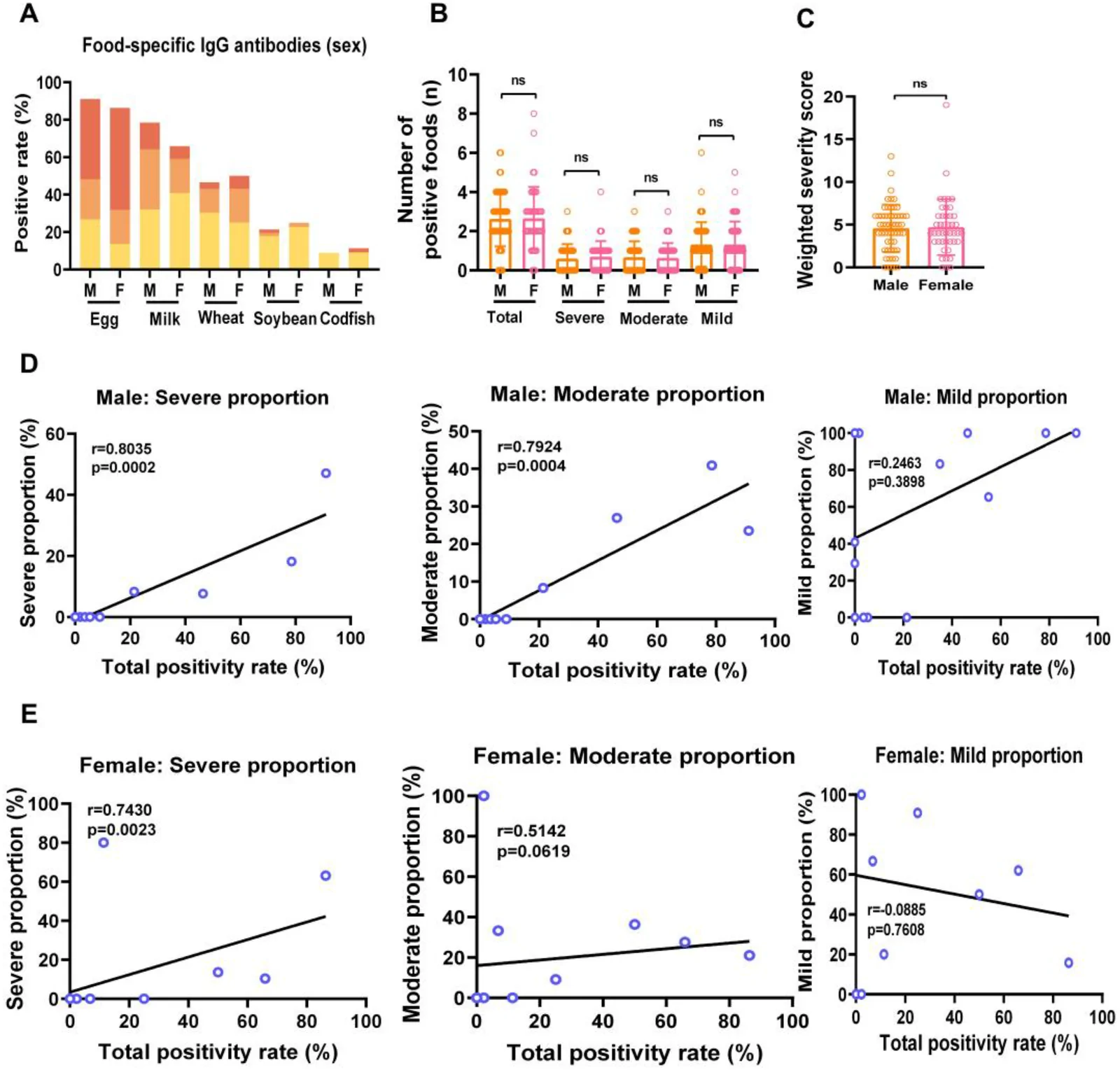
Sex-based comparison of food-specific IgG positivity rates and severity distribution. **(A)** Sex-stratified distribution of mild, moderate, and severe positivity for five major foods (egg, milk, wheat, soybean, and codfish). Horizontal stacked bar chart showing total positivity rate with color-coded segments indicating the proportions of mild, moderate, and severe positive cases for each food. **(B)** Comparison of positive food counts per child between male and female groups, including total number of positive foods and the numbers of severe, moderate, and mild positive foods. P values are shown above each pair of bars. **(C)** Comparison of the weighted severity score per child between male and female children. P values was shown above each pair of bars. **(D, E)** Correlations between total positivity rate and the proportion of mild, moderate, and severe positive cases among all positive cases for each food, shown separately for male **(D)** and female **(E)** children. Each dot represents one food item. Spearman correlation coefficients (r) and P values are shown in each panel.

Regarding food-specific IgG response intensity stratification, the distributions of mild, moderate, and severe positivity for egg, milk, and wheat did not differ significantly between sexes (egg: P = 0.326; milk: P = 0.175; wheat: P = 0.684). Furthermore, no significant differences were observed between male and female children in the total number of positive foods, the numbers of positive foods at each severity grade, or the weighted positivity scores (P > 0.05) (**Supplementary Table S3**; **Figures 2B, C**). These findings indicate that sex has no significant impact on either the positivity rate or the food-specific IgG response intensity distribution in children from this region.

To further explore sex-related differences in the correlation patterns between food positivity rate and food-specific IgG response intensity, Spearman correlation analyses were performed separately for male and female children. The total positivity rate of each of the 14 foods was used as the x-axis, and the proportions of severe, moderate, and mild positive cases among all positive cases for each food were used as the y-axes. In males, total positivity rate was significantly positively correlated with both severe proportion (r = 0.8035, P = 0.0002) and moderate proportion (r = 0.7924, P = 0.0004), but not with mild proportion (r = 0.2463, P = 0.3898) (**Figure 2D**). In females, total positivity rate was significantly positively correlated only with severe proportion (r = 0.7430, P = 0.0023), and not with moderate (r = 0.5142, P = 0.0619) or mild (r = -0.0985, P = 0.7608) proportions (**Figure 2E**).

In summary, while no significant sex differences were observed in food positivity rates or food-specific IgG response intensity distributions, the correlation patterns between positivity rate and moderate-to-severe immune responses differed by sex: in males, both moderate and severe immune responses correlated with positivity rate; in females, only severe immune responses correlated.

### Age does not affect food positivity rates, but the severity of immune responses shows a decreasing trend

To explore the influence of age on food-specific IgG distribution, the 100 children were divided into three groups: <5 years (n = 20), 5-9 years (n = 50), and ≥10 years (n = 30). The most common food items in all age groups were egg, milk, and wheat. The positivity rates for egg were 95.0%, 88.0%, and 86.7%, respectively, with no statistically significant difference among groups (χ^2^ = 1.084, P = 0.582). For milk, the rates were 90.0%, 68.0%, and 70.0% (χ^2^ = 4.362, P = 0.113). For wheat, the rates were 55.0%, 44.0%, and 50.0% (χ^2^ = 0.941, P = 0.625). No significant differences were found for soybean (Fisher’s exact test, P = 0.322) or codfish (Fisher’s exact test, P = 0.806) across age groups. The remaining foods showed sporadic positivity in all age groups, with rates ≤5.0% (**Supplementary Table S4**; **Figure 3A**).

**Figure 3:**
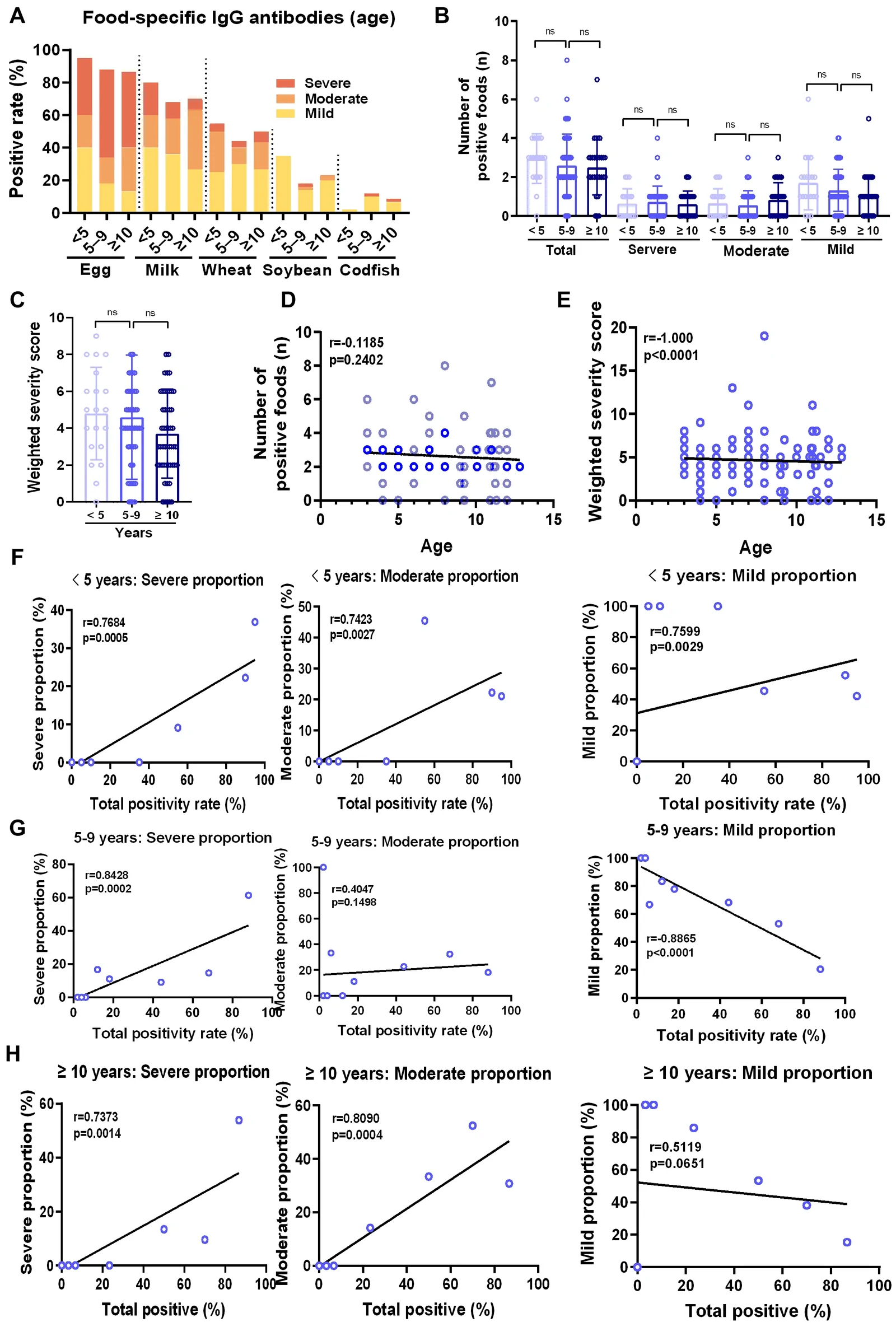
Age-based comparison of food-specific IgG positivity rates and severity distribution. **(A)** Age-stratified distribution of positivity rates for five major foods (egg, milk, wheat, soybean, and codfish). Horizontal stacked bars show total positivity rates with color-coded segments indicating the proportions of mild, moderate, and severe positive cases for each food in each age group. **(B)** Comparison of positive food counts per child among the three age groups (<5 years, 5-9 years, and ≥10 years), including total number of positive foods and the numbers of severe, moderate, and mild positive foods. P values are shown above each panel. **(C)** Comparison of weighted severity scores per child among the three age groups. P values are shown. **(D)** Correlation between age and the number of positive foods per child. Each dot represents one child. Spearman correlation coefficient (r) and P value are shown. **(E)** Correlation between age and weighted severity score per child. Each dot represents one child. Spearman correlation coefficient (r) and P value are shown. **(F–H)** Correlations between total positivity rate and the proportions of mild, moderate, and severe positive cases among all positive cases for each food, shown separately for the <5 years **(F)**, 5-9 years **(G)**, and ≥10 years **(H)** age groups. Each dot represents one food item. Spearman correlation coefficients (r) and P values are shown in each panel.

Regarding food-specific IgG response intensity stratification, the distributions of mild, moderate, and severe positivity for egg, milk, and wheat did not differ significantly among age groups (Fisher’s exact test: egg, P = 0.326; milk, P = 0.282; wheat, P = 0.779) (**Supplementary Table S5**; **Figure 3A**). The severe positivity rate for egg showed an increasing trend with age (35.0%, 54.0%, and 46.7%), though without statistical significance. For milk, mild reactions predominated in the <5 years group (50.0%), while severe cases were lower in the ≥10 years group (6.7%), suggesting a possible alleviation of food-specific IgG response intensity with increasing age (**Supplementary Table S5**; **Figure 3A**). No significant differences were found among age groups in the total number of positive foods, the numbers of mild, moderate, or severe positive foods, or the weighted positivity scores (all P > 0.05) (**Figures 3B, C**). When age was analyzed as a continuous variable, the total number of positive foods showed no significant correlation with age (r = -0.1185, P = 0.2402) (**Figure 3D**), but the weighted positivity score was significantly negatively correlated with age (r = -1.000, P < 0.0001) (**Figure 3E**), suggesting a decreasing trend in overall food-specific IgG response intensity with age.

To further explore the association between food positivity rate and food-specific IgG response intensity within each age group, Spearman correlation analyses were performed separately for the three age groups. In the <5 years group, total positivity rate was significantly positively correlated with severe proportion (r = 0.7684, P = 0.0005), moderate proportion (r = 0.7423, P = 0.0027), and mild proportion (r = 0.7599, P = 0.0029). This indicates that in young children, higher overall food-specific IgG rates are associated with increased proportions of all grades of immune responses (**Figure 3F**). In the 5-9 years group, total positivity rate was significantly positively correlated with severe proportion (r = 0.8428, P = 0.0002), not correlated with moderate proportion (r = 0.4047, P = 0.1498), and significantly negatively correlated with mild proportion (r = -0.8865, P < 0.0001), suggesting that foods with high positivity rates in this age group tend to associate with severe reactions while mild reactions decrease (**Figure 3G**). In the ≥10 years group, total positivity rate was significantly positively correlated with both severe (r = 0.7373, P = 0.0014) and moderate (r = 0.8098, P = 0.0004) proportions, and showed a borderline positive correlation with mild proportion (r = 0.5119, P = 0.0651) (**Figure 3H**).

In summary, age does not significantly affect food positivity rates, but food-specific IgG response intensity tends to decrease with age. The correlation patterns between food positivity rate and food-specific IgG response intensity vary across age groups: in the <5 years group, all three severity grades (mild, moderate, and severe) positively correlated with positivity rate; in the 5-9 years group, only severe positively correlated, while mild showed a negative correlation; in the ≥10 years group, both severe and moderate positively correlated. These findings suggest that the immune response pattern to food antigens may undergo dynamic changes with age.

### Deficiency-excess complex pattern shows the strongest association with egg-milk co-positivity

After consolidation, the four most frequent clinical symptom categories among the 100 children were: cough-related (25 cases), nasal diseases (20 cases), tic disorder/chronic tic disorders (19 cases), and deficiency-excess complex pattern (9 cases) (**Supplementary Table S6**; **Figure 4A**). Comparison of single-food positivity rates across these four symptom groups showed that only milk positivity differed significantly among groups (χ^2^ = 7.862, P = 0.049), with the cough group having the highest rate (92.0%), significantly higher than the tic disorder/chronic tic disorders group (63.2%) and the nasal diseases group (70.0%). No significant differences were observed for egg, wheat, or soybean across groups (P > 0.05). Regarding food spectrum breadth, the nasal diseases group showed a numerically higher positivity rate for codfish (20.0%) compared to the cough group (8.0%) and the tic disorder/chronic tic disorders group (10.5%) (**Supplementary Table S6**; **Figure 4A**), suggesting a slightly broader food spectrum in this group.

**Figure 4:**
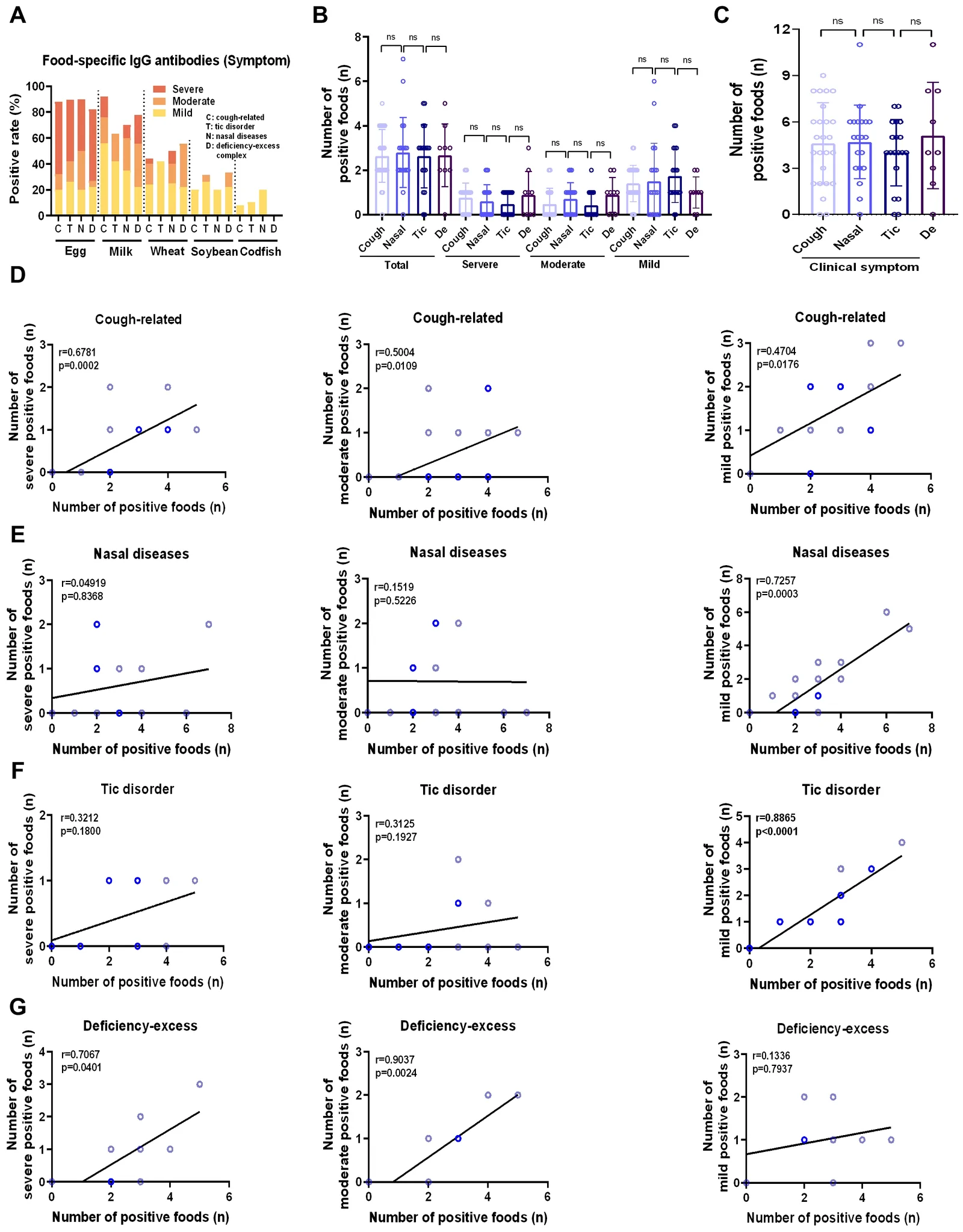
Clinical symptom-based comparison of food-specific IgG positivity rates and severity distribution. **(A)** Positivity rates of five major foods (egg, milk, wheat, soybean, and codfish) across four clinical symptom groups: cough-related, nasal diseases, tic disorder/chronic tic disorders, and deficiency-excess complex pattern. Horizontal stacked bars show total positivity rates with color-coded segments indicating the proportions of mild, moderate, and severe positive cases for each food in each clinical symptom group. **(B)** Comparison of positive food counts per child among the four symptom groups, including total number of positive foods and the numbers of severe, moderate, and mild positive foods. P values are shown above each panel. **(C)** Comparison of weighted severity scores per child among the four symptom groups. P values are shown. **(D–G)** Correlations between the number of positive foods per child and the numbers of severe, moderate, and mild positive foods, shown separately for cough-related **(D)**, nasal diseases **(E)**, tic disorder/chronic tic disorders **(F)**, and deficiency-excess complex pattern **(G)**. Each dot represents one child. Spearman correlation coefficients (r) and P values are shown in each panel.

In terms of food-specific IgG response intensity stratification, the distributions of mild, moderate, and severe positivity for egg, milk, and wheat did not differ significantly among the four symptom groups (Fisher’s exact test, P > 0.05) (**Supplementary Table S7**; **Figure 4A**). No significant differences were found among the four groups in the total number of positive foods, the numbers of positive foods at each severity grade, or the weighted positivity scores (P > 0.05) (**Figures 4B, C**), suggesting that the overall intolerance burden was largely balanced across groups.

To explore the association between food positivity rate and food-specific IgG response intensity within each symptom group, Spearman correlation analyses were performed separately for the four groups. In the cough-related group, the total number of positive foods was positively correlated with the numbers of severe (r = 0.6781, P = 0.0002), moderate (r = 0.5004, P = 0.0109), and mild (r = 0.4704, P = 0.0175) positive foods (**Figure 4D**). In the nasal diseases group, the total number of positive foods was positively correlated only with the number of mild positive foods (r = 0.7257, P = 0.0003), and not with severe (r = -0.0492, P = 0.8368) or moderate (r = 0.1519, P = 0.5226) positive foods (**Figure 4E**). The tic disorder/chronic tic disorders group showed a similar pattern, with a positive correlation only with mild positive foods (r = 0.8865, P < 0.0001), and no significant correlations with severe (r = 0.3212, P = 0.1600) or moderate (r = 0.3125, P = 0.1927) positive foods (**Figure 4F**). The deficiency-excess complex pattern group, however, showed a different pattern: the total number of positive foods was positively correlated with severe (r = 0.7067, P = 0.0401) and moderate (r = 0.9037, P = 0.0024) positive foods, but not with mild positive foods (r = -0.1336, P = 0.7937) (**Figure 4G**), suggesting that in this group, an increase in food-specific IgG breadth is associated with a concurrent increase in moderate-to-severe positive foods.

To identify common multi-food food-specific IgG combination patterns among different clinical phenotypes, the Apriori algorithm was applied for association rule mining, with the analysis direction set as clinical symptoms pointing to food-specific IgG combinations. With a minimum support of 6%, a minimum confidence of 50%, and a lift >1.5, four strong association rules were identified. The strongest rule was deficiency-excess complex pattern with egg-milk co-positivity (support 7.0%, confidence 77.8%, lift 6.09); the second was deficiency-excess complex pattern with egg moderate-to-severe positivity (support 6.0%, confidence 66.7%, lift 5.68) (**Table 2**). Cough-related symptoms with egg-milk co-positivity (support 16.0%, confidence 64.0%, lift 2.54) and tic disorder/chronic tic disorders with egg moderate-to-severe positivity (support 10.0%, confidence 52.6%, lift 2.15) also showed relatively strong associations. The deficiency-excess complex pattern dominated all rules, with a lift (6.09) markedly higher than that of other symptom groups (**Table 2**). Although the nasal diseases group did not yield an independent strong rule, its egg-milk co-positivity rate was 70.0% (14/20) at the descriptive level (**Table 2**). The reverse direction (food-specific IgG combinations pointing to clinical symptoms) did not identify any strong association rules meeting the criteria under the same parameters, with all confidences below 50%.

**Table 2:** Strong association rules between clinical symptoms and food intolerance combinations identified by Apriori algorithm.

Rank	Antecedent (symptom)	Consequent (food combination)	Support (%)	Confidence (%)	Lift
1	Deficiency-excess complex pattern	Egg and milk co-positivity	7.0	77.8	6.09
2	Deficiency-excess complex pattern	Egg moderate-to-severe positivity	6.0	66.7	5.68
3	Cough-related symptoms	Egg and milk co-positivity	16.0	64.0	2.54
4	Tic disorder/chronic tic disorders	Egg moderate-to-severe positivity	10.0	52.6	2.15

Minimum support = 6%, minimum confidence = 50%. Lift > 1.5 was considered as a meaningful positive association. The analysis direction was set as clinical symptom → food intolerance combination.

These results suggest that in this study population, single or combined food-specific IgG has weak specificity for particular clinical symptoms, and the symptom-to-food-specific IgG combination direction may offer greater clinical utility. Clinicians encountering children predominantly presenting with deficiency-excess complex pattern or cough-related symptoms may consider egg and milk as priority foods for dietary intervention.

## Discussion

The clinical value of food-specific IgG testing in the management of chronic pediatric conditions has drawn increasing attention (4), yet regional epidemiological data and the complex association patterns between food-specific IgG and specific symptoms remain to be elucidated. Through systematic analysis of 14 food-specific IgG antibodies in 100 children under 14 years of age from the Zhuhai area, combined with association rule mining, this study revealed the predominant food-specific IgG profiles, demographic determinants, and their relationships with clinical symptoms in this population. The involved factors-including intestinal barrier integrity, microbiota composition, and inflammatory cytokines-have not been experimentally validated.

The predominance of egg, milk, and wheat as the top three food-specific IgG positivities in this study is consistent with multiple domestic epidemiological surveys. A large-sample study from Hebei Province (31,312 cases) similarly identified egg as the food with the highest positivity rate (57.13%) (7). A study from Saudi Arabia involving 75 children with allergic diseases found that dairy products, gluten-containing cereals, and eggs were the three most common food-specific IgG-positive items (9). Egg and milk consistently rank as the most frequently positive foods across various studies, which may be attributed to early and sustained exposure to these two foods during childhood. The underlying mechanism involves the strong antigenicity and resistance to digestion of ovalbumin, casein, and gliadin present in these foods (10, 11). When the intestinal barrier is not fully mature in children, incompletely digested protein macromolecules can readily penetrate the mucosa and trigger IgG-mediated delayed immune responses.

Notably, in Zhuhai, a coastal city, the positivity rates for seafood (shrimp and crab) were extremely low (1.0% each). This contrast between high seafood consumption and low IgG positivity, which stands in stark contrast to the relatively high prevalence of IgE-mediated seafood allergy in this region (12), further confirms the fundamental mechanistic difference between IgG-mediated FI and IgE-mediated immediate-type allergy. FI reflects immune responses to long-term cumulative exposure, whereas allergy is associated with specific sensitization. This finding suggests that clinicians should not implement blanket dietary restrictions based solely on regional dietary habits, in order to avoid unnecessary nutritional imbalance. And our findings do not provide a reference for IgG baseline levels in the general pediatric population nor should they be extrapolated to healthy children.

Our results demonstrated that total food positivity rate was positively correlated with severe proportion and negatively correlated with mild proportion, indicating that foods with higher positivity rates are more likely to elicit severe immune responses. This pattern was validated at both the food level and the individual level, suggesting that the consistency between positivity rate and severe proportion may represent a fundamental characteristic of food-specific IgG. This finding differs markedly from IgE-mediated allergic reactions, in which no such linear relationship exists between specific sensitization and symptom severity-minute amounts of allergen can trigger life-threatening reactions (13, 14). In contrast, IgG-mediated intolerance exhibits a cumulative effect, whereby foods with greater exposure and higher positivity rates are associated with increased probability of moderate-to-severe reactions (15). This difference may stem from the distinct immunological mechanisms underlying the two types of responses: IgE reactions depend on sensitization status, whereas IgG reactions are related to cumulative antigen load. Food-specific IgE levels were not measured in this study; therefore, we are unable to directly assess whether sex differences also exist in IgE-mediated allergic pathways. It should be noted that this study focused on food-specific IgG positivity, which is fundamentally different from IgE-mediated immediate-type allergy in terms of immune mechanisms. Future studies could measure both food-specific IgG and IgE, along with sex hormone levels, to more comprehensively elucidate the role of sex in food-related immune responses.

The observed decrease in immune response intensity with age does not contradict the cumulative effect of IgG-mediated intolerance, as these two phenomena operate at different levels. The cumulative effect describes a cross-sectional comparison across foods within the same age group: foods with higher exposure and positivity rates are associated with a higher probability of moderate-to-severe reactions-a horizontal dose–response relationship. The age-related decline, in contrast, reflects longitudinal developmental changes in the individual, including maturation of the intestinal barrier, improved digestive capacity, and establishment of oral immune tolerance, which collectively raise the threshold for immune responses and reduce overall response intensity. These two patterns can be understood as follows: at younger ages, the intestinal barrier and immune regulatory functions are relatively immature, making severe reactions more likely under similar exposure levels; with increasing age, as the barrier strengthens, even if cumulative exposure continues to increase, response intensity declines due to tolerance development. The finding that food positivity rates do not change with age while response intensity decreases supports this interpretation.

The decision to measure total IgG rather than the IgG4 isotype was based on the following considerations (1): Clinical applicability. The commercial ELISA kit used in this study detects total food-specific IgG, which is a commonly used test for food intolerance in Chinese clinical practice. Measuring total IgG allows our results to be directly comparable with the existing clinical testing framework (2). Comprehensive coverage of IgG subclasses. Different foods elicit distinct IgG subclass responses. IgG responses to egg and wheat are predominantly IgG4, whereas those to milk involve multiple subclasses including IgG2, IgG3, and IgG4. Measuring total IgG avoids the potential risk of missing subclass-mediated reactions that would occur if only IgG4 were measured (3). Alignment with the study phase. The relationship between IgG4 and symptom remission or prognosis pertains to more in-depth mechanistic and intervention research. As this study is a regional epidemiological survey, its primary aim was to characterize the population-level IgG profiles and their associations with symptoms. Detailed analysis of IgG4 subclasses is better addressed in future studies. In addition, IgG4 accounts for only 3%-6% of total IgG in healthy individuals, and the European Academy of Allergy and Clinical Immunology (EAACI) and other international societies do not recommend the use of food-specific IgG4 testing for routine diagnosis, as its clinical significance remains controversial. Therefore, measuring total IgG represents a more appropriate and pragmatic choice at this stage of research.

The individual-level findings of this study have clinical relevance. Children with a greater number of positive foods showed corresponding increases in severe food counts and weighted scores, suggesting that clinical assessment should not focus solely on the positivity of individual foods, but rather on the overall burden reflected by the total number of positive foods. This pattern is consistent with the food-level analysis and provides a reference for clinical evaluation.

In terms of sex differences, both moderate and severe immune responses correlated with food positivity rate in males, whereas only severe responses correlated in females. This pattern suggests that sex may influence the distribution pattern of food-specific IgG response intensity rather than the positivity rate itself. Previous studies have shown that estrogen enhances B-cell antibody production and Th2-type immune responses, while androgens tend to suppress humoral immunity (16, 17). Thus, the weaker association at the moderate response level in females may reflect sex differences in B-cell activation thresholds and immunoregulation. However, given the limited number of relevant reports, this hypothesis requires further investigation. Clinically, this finding suggests that for the same positive food, the probability of moderate-to-severe reactions may differ between male and female children, and the priority of dietary intervention may need to be determined with consideration of sex.

Regarding age, no significant differences in positivity rates were observed across age groups, but weighted positivity scores were negatively correlated with age, with food-specific IgG response intensity, showing a decreasing numerical trend with age. Previous studies have shown that milk- and egg-specific IgG antibody titers peak between 5 months and 1 year of age and gradually decline to low levels in adulthood (18). Similar observations have been made in children with natural resolution of peanut allergy, where peanut-specific IgG4 levels decreased from 1 to 10 years of age (19). This suggests that food-specific IgG levels tend to decrease with age, which may be associated with gradual maturation of the intestinal barrier and the establishment of oral immune tolerance (20). Positive in the <5 years group, negative in the 5-9 years group, and borderline positive in the ≥10 years group. This changing pattern aligns with the developmental dynamics of food-related immune responses in children. During the preschool period, the intestinal barrier is not yet fully mature and oral immune tolerance has not been completely established. Food antigen exposure tends to induce mild immune responses rather than directly triggering moderate-to-severe reactions. Consequently, foods with higher positivity rates also show higher mild proportions. During early school age, sustained antigen exposure drives a cascade amplification of immune responses from mild toward moderate-to-severe, resulting in a decreased mild proportion for highly positive foods. In middle-to-late school age, as the intestinal barrier matures and oral immune tolerance gradually develops, overall immune response intensity declines, severe responses subside, and the mild proportion rises again. This transition from positive to negative to borderline positive correlation corresponds to the complete trajectory of initial sensitization, progressive amplification, and eventual tolerance resolution in the developmental course of childhood food-related immune responses.

Association rule mining in this study revealed co-occurrence patterns between clinical symptoms and multi-food-specific IgG combinations. The strongest association was found between deficiency-excess complex pattern and egg-milk co-positivity (lift 6.09), indicating that 77.8% of children with this pattern had concurrent egg and milk intolerance. In traditional Chinese medicine theory, the deficiency-excess complex pattern corresponds to a pathogenesis of spleen deficiency with dampness retention and intermingled deficiency and excess, characterized by impaired transportation and transformation leading to accumulation of dampness with concurrent vital energy deficiency. From a modern medical perspective, egg and milk are the earliest introduced and most frequently consumed food antigens in infancy. Their large molecular weight and resistance to digestion facilitate their passage through the intestinal mucosa into the circulation when intestinal barrier function is compromised (4). We speculate that children with the deficiency-excess complex pattern may exhibit alterations in intestinal barrier function, although this mechanism was not directly tested in the present study. Children with the deficiency-excess complex pattern may have greater intestinal permeability than those in other groups, resulting in more concentrated immune responses to egg and milk. The dominance of this rule across all identified associations suggests that children with the deficiency-excess complex pattern may represent a subgroup characterized by impaired intestinal barrier function with multi-food sensitization, and the clinical benefit of egg and milk avoidance may be most pronounced in this group.

The confidence of cough-related symptoms with egg-milk co-positivity was 64.0% (lift 2.54), indicating that nearly two-thirds of children with cough had concurrent egg and milk intolerance. From a gut-lung axis perspective, food antigens crossing the intestinal barrier can transport immune complexes via the circulation to the respiratory mucosa. Animal studies have demonstrated that gut microbiota dysbiosis can alter the pulmonary immune microenvironment and enhance airway responsiveness to allergens (21, 22). Our findings provide population-level support for this theory: the high co-occurrence of egg and milk positivity in children with cough suggests that exposure to these two food antigens may be an important trigger for intestinal immune dysregulation affecting the respiratory tract. Previous case reports have shown that cow’s milk protein intolerance can present solely as recurrent respiratory tract infections, with marked improvement after dietary elimination (23), and our study further supports this clinical phenomenon at the population level.

This study has several limitations. First, we acknowledge that existing literature indicates that food-specific IgG positivity does occur in healthy children, although the reported rates vary considerably depending on age, region, and detection methods. Meanwhile, the positivity rates observed in our study-89.0% for egg and 73.0% for milk-are broadly comparable to those reported in similar domestic studies conducted in China (e.g., 81.50% in Qingdao and 57.13% in Hebei, suggesting that our findings may largely reflect regional dietary exposure patterns. However, in the absence of concurrent healthy controls, we are unable to determine whether these positivity rates are abnormally elevated. Second, the relatively small sample size (100 cases) limits statistical power in some subgroups, and the association rule mining results should be interpreted with caution. Third, the single-center retrospective design may introduce selection bias and limit generalizability of the findings. Fourth, deficiency-excess complex pattern” group was the limited sample size-each individual subtype had fewer than 5 cases, which would not have permitted meaningful association rule analysis. We acknowledge this limitation and have explicitly marked this grouping as exploratory in the revised manuscript, noting that the distinct immune profiles associated with each syndrome subtype warrant further investigation in larger samples. Fifth, food-specific IgG testing reflects exposure levels only; causal relationships with clinical symptoms cannot be inferred from this study, and the observed associations require validation through prospective cohort studies or dietary intervention trials. Sixth, association rule mining is an exploratory data-driven approach, and the rules identified carry inherent stochasticity and require validation in independent samples.

Does elimination of IgG-positive foods improve clinical symptoms? Are IgG levels reversible after dietary intervention? Is this testing approach clinically actionable? We have clearly stated that these questions are beyond the scope of the present study and require prospective randomized controlled intervention studies to address.

In summary, this study revealed that egg, milk, and wheat are the predominant IgG‑positive foods among children in the Zhuhai area, and that food positivity rate is positively correlated with the proportion of severe immune responses it triggers. Sex and age did not significantly affect positivity rates, but the correlation patterns between positivity rate and food-specific IgG response intensity differed by sex, and food-specific IgG response intensity tended to decrease with age. Association rule mining further identified that the deficiency-excess complex pattern showed the strongest co-occurrence with egg-milk co-positivity. It should be acknowledged that only 9 patients were included in the mixed deficiency-excess syndrome group, rendering the sample size exceedingly small. Given that association rule mining is an exploratory method, the observed patterns are inherently susceptible to random variation and are substantially contingent upon the distribution of the sample. As such, these results do not possess statistical stability, preclude any extrapolation, and serve merely as preliminary signals for hypothesis formulation in subsequent investigations. These findings may provide a reference for dietary intervention in children with food-specific IgG in this region; however, their causal relationships and clinical utility require further validation through prospective studies. It should be noted that the present study assessed food-specific IgG antibodies; accordingly, a positive result signifies an immunological response to food exposure and should not be interpreted as a definitive diagnosis of pathological food intolerance. Subsequent investigations, building upon an enlarged cohort, could employ a “same disease with heterogeneous syndromes” study design-for instance, subclassifying chronic cough into distinct traditional Chinese medicine (TCM) pattern classifications-thereby enabling a more accurate delineation of the associations between specific syndrome types and corresponding immunological signatures. Future studies with larger sample sizes conduct stratified analyses by specific syndrome subtype. Future studies include healthy children matched for age, sex, and dietary patterns as controls, in order to distinguish disease-related immune abnormalities from physiological IgG patterns associated with dietary exposure. This is a single-center, exploratory, uncontrolled study, and that the findings require validation in future studies with healthy control groups.

## Materials and methods

### Study participants

This retrospective study included 100 children who attended Zhuhai Hospital of Traditional Chinese Medicine between January 2024 and December 2025. The cohort comprised 56 males and 44 females, with ages ranging from 2.0 to 12.8 years (mean age 7.2 ± 2.8 years). In this study, children aged 2.0 to 12.8 years were divided into three groups: <5 years, 5-9 years, and ≥10 years. The rationale for this classification is as follows (1): the <5 years group represents the preschool stage, during which the intestinal barrier and immune system are not yet fully mature, making this a high-risk period for FI (2); the 5-9 years group corresponds to early school age, when dietary patterns become more stable and antigen exposure is sustained, with chronic symptoms tending to emerge; and (3) the ≥10 years group represents middle-to-late school age, when immune function gradually matures and FI intensity tends to decrease. This grouping takes into account both developmental stage characteristics and the natural distribution of the sample, and is consistent with the age stratification logic used in similar domestic studies. In addition, the sample size distribution across the three age groups was relatively balanced (20, 50, and 30 cases), providing basic statistical power for the subsequent analyses.

The final enrollment of 100 children. This figure has been added to the supplementary materials as **Supplementary Figure S1**.

Inclusion criteria were (1): age ≤ 14 years; and (2) clinical diagnosis of allergic rhinitis, chronic cough, or tic disorder, consistent with national clinical guidelines.

Exclusion criteria included (1): severe underlying conditions such as congenital heart disease, autoimmune disorders, or malignancies (2); use of corticosteroids or immunosuppressants within one month prior to testing (3); concurrent acute infection; and (4) missing key clinical data.

This study complied with medical ethical requirements, and informed consent was obtained from the guardians of all participating children.

### Serum collection and preparation

Fasting venous blood samples (3-5 mL) were collected from all children in the morning using dry vacuum tubes without anticoagulants. Blood samples were allowed to clot at room temperature for 30 minutes, followed by centrifugation at 3000 × g for 10 minutes to separate serum. Serum samples were stored at -20 °C until analysis, and repeated freeze-thaw cycles were avoided.

### Detection of food-specific IgG antibodies

Serum levels of specific IgG antibodies against 14 foods (milk, egg, wheat, soybean, corn, tomato, rice, mushroom, codfish, shrimp, crab, beef, lamb, and pork) were measured using enzyme-linked immunosorbent assay (ELISA). The kits were purchased from Biomerica, USA (Catalog No. MB00077). All procedures were performed according to the manufacturer’s instructions. Serum samples were diluted 1:100 and added to microplates coated with different food antigens, followed by incubation at 37 °C for 30 minutes. After three washes, enzyme-labeled secondary antibodies were added and incubated at 37 °C for 30 minutes. After five washes, substrate solution was added and incubated in the dark for 15 minutes. The reaction was terminated, and optical density was read at 450 nm.

### The positive classification of individual antibody responses

The IgG antibody concentrations (U/mL) was classified into three tiers according to the reference ranges provided by the food-specific IgG antibody detection kit (Biomerica, USA, MB00077). This classification system targets the food-specific IgG response intensity of an individual child to a specific food, based on the serum food-specific IgG antibody concentration (U/mL), and is used to determine the severity of intolerance at the individual level. The specific criteria are as follows: negative (grade 0), <50 U/mL; mild positive (+1), 50 to <100 U/mL; moderate positive (+2), 100 to <200 U/mL; severe positive (+3), ≥200 U/mL. Grade +1 and above were considered positive. This parameter reflects only the quantitative level of antibody detection and should not be assigned pathological meaning beyond the biochemical measurement itself.

### Clinical symptom and traditional Chinese medicine syndrome classification

Clinical diagnoses and symptom labels (44 in total) were collected from all children. After consolidation, the four most frequent symptom categories were: cough-related symptoms (25 cases), including cough, bronchitis, acute bronchitis, cough variant asthma, and *Mycoplasma pneumoniae* acute bronchitis; nasal diseases (20 cases), including allergic rhinitis, sinusitis, nasal obstruction, chronic sinusitis, nasal septum deviation, and acute rhinitis with nasal congestion; tic disorder/chronic tic disorders (19 cases); and deficiency-excess complex pattern (9 cases), including lung-spleen deficiency pattern. All TCM syndrome diagnoses in this study were based on the criteria defined in TCM Pediatrics (10th edition) and the Criteria for Diagnosis and Therapeutic Effects of TCM Diseases and Syndromes. Syndrome classification was independently performed by two pediatric TCM physicians with attending physician or higher qualifications, based on original outpatient medical records. In cases of disagreement, a chief TCM physician made the final determination. All syndrome data were double-entered and verified.

Regarding diagnostic consistency: Due to the retrospective design of this study, syndrome differentiation was based on pre-existing medical records; therefore, Kappa statistics were not calculated. For future prospective studies, we plan to adopt standardized syndrome differentiation scales and Kappa consistency testing to further improve the reproducibility and objectivity of syndrome classification.

### Statistical analysis

Statistical analyses were performed using SPSS 26.0 and Python 3.8. Categorical variables were presented as counts (n) and percentages (%), and group comparisons were performed using the χ^2^ test or Fisher’s exact test when expected frequencies were below 5. Continuous variables were expressed as mean ± standard deviation, with comparisons between two groups using the t-test or Mann-Whitney U test, and among multiple groups using the Kruskal-Wallis test. Correlations were assessed using Spearman’s rank correlation coefficient.

Association rule mining was performed using the Apriori algorithm, with the analysis direction set as clinical symptoms pointing to food-specific IgG combinations. The minimum support was set at 6%, minimum confidence at 50%, and rules with a lift >1.5 were considered strong. A P-value <0.05 was considered statistically significant.

For multiple group comparisons involving more than two categories, Bonferroni correction was applied to control the family-wise error rate. A corrected P-value <0.05 was considered statistically significant. Given the exploratory nature of association rule mining and the use of multiple filtering criteria (support, confidence, and lift), no additional correction for multiple comparisons was applied.

## Data Availability

The original contributions presented in the study are included in the article/**Supplementary Material**. Further inquiries can be directed to the corresponding authors.
