# Supplementary material for: Association rule analysis of food-specific IgG and clinical symptoms in pediatric patients
Source: Front Immunol. 2026 Sep 16;17:1940230. doi: 10.3389/fimmu.2026.1940230 (PMC13623606; doi:10.3389/fimmu.2026.1940230)
Supplement: Supplementary file 1 [file DataSheet1.doc]

**Figure S1. Flowchart of patient recruitment and enrollment.**

**Table S1. Prevalence and severity grading of food-specific IgG antibodies to 14 foods (n=100)**

| Rank | Food | Positive n (%) | Mild (+1) | Moderate (+2) | Severe (+3) |
| --- | --- | --- | --- | --- | --- |
| 1 | Egg | 89 (89.0) | 21 | 20 | 48 |
| 2 | Milk | 73 (73.0) | 36 | 26 | 11 |
| 3 | Wheat | 48 (48.0) | 28 | 15 | 5 |
| 4 | Soybean | 23 (23.0) | 20 | 2 | 1 |
| 5 | Codfish | 10 (10.0) | 9 | 0 | 1 |
| 6 | Chicken | 5 (5.0) | 4 | 1 | 0 |
| 7 | Beef | 4 (4.0) | 3 | 1 | 0 |
| 8 | Rice | 4 (4.0) | 4 | 0 | 0 |
| 9 | Tomato | 3 (3.0) | 3 | 0 | 0 |
| 10 | Pork | 1 (1.0) | 0 | 1 | 0 |
| 11 | Corn | 1 (1.0) | 1 | 0 | 0 |
| 12 | Mushroom | 1 (1.0) | 1 | 0 | 0 |
| 13 | Crab | 1 (1.0) | 1 | 0 | 0 |
| 14 | Shrimp | 1 (1.0) | 1 | 0 | 0 |

**Note:** According to the positivity rate, the 14 foods were classified into three tiers: high-positivity tier (≥40%): egg, cow's milk, wheat; moderate-positivity tier (10%–25%): soybean, codfish; low-positivity tier (≤5%): chicken, beef, rice, tomato, pork, corn, mushroom, crab, shrimp.

**Table S2. Sex-based comparison of food-specific IgG positivity rates**

| Food | Male (n=56) n (%) | Female (n=44) n (%) | χ² | P value |
| --- | --- | --- | --- | --- |
| Egg | 51 (91.1) | 38 (86.4) | 0.558 | 0.455 |
| Milk | 44 (78.6) | 29 (65.9) | 2.005 | 0.157 |
| Wheat | 26 (46.4) | 22 (50.0) | 0.126 | 0.723 |
| Soybean | 12 (21.4) | 11 (25.0) | 0.178 | 0.673 |
| Codfish | 5 (8.9) | 5 (11.4) | 0.158 | 0.691 |
| Chicken† | 2 (3.6) | 3 (6.8) | – | 0.655 |
| Beef† | 1 (1.8) | 2 (4.5) | – | 0.589 |
| Pork† | 0 (0.0) | 1 (2.3) | – | 0.440 |

*Fisher's exact test was applied due to expected cell frequencies <5.

**Table S3. Sex-stratified distribution of severity grades for three major food-specific IgG antibodies**

| **Food** | **Sex** | **Mild n (%)** | **Moderate n (%)** | **Severe n (%)** | **P value*** |
| --- | --- | --- | --- | --- | --- |
| Egg | Male | 15 (26.8) | 12 (21.4) | 24 (42.9) | 0.326 |
|  | Female | 6 (13.6) | 8 (18.2) | 24 (54.5) |  |
| Milk | Male | 18 (32.1) | 18 (32.1) | 8 (14.3) | 0.175 |
|  | Female | 18 (40.9) | 8 (18.2) | 3 (6.8) |  |
| Wheat | Male | 17 (30.4) | 7 (12.5) | 2 (3.6) | 0.684 |
|  | Female | 11 (25.0) | 8 (18.2) | 3 (6.8) |  |
| Soybean | Male | 10 (17.9) | 1 (1.8) | 1 (1.8) | 1.000 |
|  | Female | 10 (22.7) | 1 (2.3) | 0 (0.0) |  |
| Codfish | Male | 5 (8.9) | 0 (0.0) | 0 (0.0) | 1.000 |
|  | Female | 4 (9.1) | 0 (0.0) | 1 (2.3) |  |

*Fisher's exact test was applied to compare the distribution of severity grades between sexes for each food.

**Table S4. Age-stratified comparison of major food-specific IgG positivity rates**

| Food | <5 years (n=20) n (%) | 5-9 years (n=50) n (%) | ≥10 years (n=30) n (%) | χ² | P value |
| --- | --- | --- | --- | --- | --- |
| Egg | 19 (95.0) | 44 (88.0) | 26 (86.7) | 1.084 | 0.582 |
| Milk | 18 (90.0) | 34 (68.0) | 21 (70.0) | 4.362 | 0.113 |
| Wheat | 11 (55.0) | 22 (44.0) | 15 (50.0) | 0.941 | 0.625 |
| Soybean | 7 (35.0) | 9 (18.0) | 7 (23.3) | – | 0.322* |
| Codfish | 2 (10.0) | 6 (12.0) | 2 (6.7) | – | 0.806* |

*Fisher's exact test was applied due to expected cell frequencies <5.

**Table S5. Age-stratified distribution of severity grades for three major food-specific IgG antibodies**

| Food | Age group | Mild n (%) | Moderate n (%) | Severe n (%) | P value* |
| --- | --- | --- | --- | --- | --- |
| Egg | <5 years | 8 (40.0) | 4 (20.0) | 7 (35.0) | 0.326 |
|  | 5-9 years | 9 (18.0) | 8 (16.0) | 27 (54.0) |  |
|  | ≥10 years | 4 (13.3) | 8 (26.7) | 14 (46.7) |  |
| Milk | <5 years | 10 (50) | 4 (20.0) | 4 (20.0) | 0.282 |
|  | 5-9 years | 18 (36.0) | 11 (22.0) | 5 (10.0) |  |
|  | ≥10 years | 8 (26.7) | 11 (36.7) | 2 (6.7) |  |
| Wheat | <5 years | 5 (25) | 5 (25) | 1 (5) | 0.779 |
|  | 5-9 years | 15 (30.0) | 5 (10.0) | 2 (4.0) |  |
|  | ≥10 years | 8 (26.7) | 5 (16.7) | 2 (6.7) |  |
| Soybean | <5 years | 7 (35) | 0 (0) | 0 (0) | 1.000 |
|  | 5-9 years | 7 (14.0) | 1 (2.0) | 1 (2.0) |  |
|  | ≥10 years | 6 (20.0) | 1 (3.3) | 0 (0) |  |
| Codfish | <5 years | 2 (10.0) | 0 (0) | 0 (0) | 1.000 |
|  | 5-9 years | 5 (10.0) | 0 (0) | 1 (2.0) |  |
|  | ≥10 years | 2 (6.7) | 0 (0) | 1 (2.0) |  |

*****Fisher's exact test. Values are presented as n (% of age-specific group). P values >0.05 for all comparisons.

**Table S6. Comparison of food-specific IgG positivity rates among major clinical symptom groups**

| Food | Cough-related (n=25) n (%) | Tic disorder (n=19) n (%) | Nasal diseases (n=20) n (%) | Deficiency-excess complex (n=9) n (%) | χ² | P value |
| --- | --- | --- | --- | --- | --- | --- |
| Egg | 22 (88.0) | 17 (89.5) | 18 (90.0) | 8 (88.9) | 0.174 | 0.982 |
| Milk | 23 (92.0) | 12 (63.2) | 14 (70.0) | 7 (77.8) | 7.862 | **0.049** |
| Wheat | 11 (44.0) | 8 (42.1) | 10 (50.0) | 5 (55.6) | 0.701 | 0.873 |
| Soybean | 5 (20.0) | 6 (31.6) | 4 (20.0) | 3 (33.3) | 1.540 | 0.673 |
| Codfish | 2 (8.0) | 2 (10.5) | 4 (20.0) | 0 (0.0) | — | 0.517* |

*Fisher's exact test was applied due to expected cell frequencies <5.

**Table S7. Clinical symptom -stratified distribution of severity grades for major food-specific IgG antibodies**

| Food | Clinical symptom | Mild n (%) | Moderate n (%) | Severe n (%) | P value* |
| --- | --- | --- | --- | --- | --- |
| Egg | Cough-related | 5 (20.0) | 3 (12.0) | 14 (56.0) | 0.724 |
|  | Tic disorder | 5 (26.3) | 3 (15.8) | 9 (47.4) |  |
|  | Nasal diseases | 4 (20.0) | 6 (30.0) | 8 (40.0) |  |
|  | Deficiency-excess complex | 2 (22.2) | 1 (4.5) | 5 (55.6) |  |
| Milk | Cough-related | 14 (56.0) | 5 (20.0) | 4 (16.0) | 0.324 |
|  | Tic disorder | 8 (42.1) | 4 (21.1) | 0 (0.0) |  |
|  | Nasal diseases | 7 (35.0) | 5 (25.0) | 2 (10.0) |  |
|  | Deficiency-excess complex | 2 (22.2) | 3 (33.3) | 2 (22.2) |  |
| Wheat | Cough-related | 6 (24.0) | 4 (16.0) | 1 (4.0) | 0.204 |
|  | Tic disorder | 8 (42.1) | 0 (0.0) | 0 (0.0) |  |
|  | Nasal diseases | 5 (25.0) | 3 (15.0) | 2 (10.0) |  |
|  | Deficiency-excess complex | 2 (22.2) | 3 (33.3) | 0 (0.0) |  |
| Soybean | Cough-related | 5 (20.0) | 0 (0) | 0 (0) | 0.569 |
|  | Tic disorder | 5 (26.3) | 1 (5.3) | 0 (0) |  |
|  | Nasal diseases | 4 (20.0) | 0 (0) | 0 (0) |  |
|  | Deficiency-excess complex | 2 (22.2) | 1 (11.1) | 0 (0) |  |
| Codfish | Cough-related | 2 (8.0) | 0 (0) | 0 (0) | 0.493 |
|  | Tic disorder | 2 (10.5) | 0 (0) | 0 (0) |  |
|  | Nasal diseases | 4 (20.0) | 0 (0) | 0 (0) |  |
|  | Deficiency-excess complex | 0 (0.0) | 0 (0.0) | 0 (0.0) |  |

*Fisher's exact test was applied to compare the distribution of severity grades among symptom groups for each food.
